# Mary

**Published:** 1904-10-15

**Authors:** R. E. Gilson


### "MARY."

I believe in delegating to the assistant (excuse me, I mean to say Mary) such details as we may, to have the operator free for that which demands his special skill and knowledge. Hence, Mary makes out the appointments, and she must be accurate. Mistakes in time, or failure on the dentist's part in keeping of appointments, have cost many a man in patients and reputation. So the assistant bears the responsibility for her employer's reputation in her accuracy or errors. And in another way, too. She is the first to greet strangers, and much depends upon impressions of neatness. And if she be neat of dress, she will be so with the office. Even in a building like this, where daily janitor service is provided, dust will creep over chairs and desks and tables after the janitors have done, and California west winds will add their quota. So dusting morning and noon will add to a neat appearance, and care of appearance betokens antiseptic care of instruments and care in operation. So much depends on neat appearance. One woman left her dentist because his nails were not clean and another changed because the drinking glass offered to her showed signs of having been used by another. One lapse may lose a patient.

### NAPKINS AND THINGS.

While speaking of cleanliness I am reminded of the way some use the aseptic doilies while operating. Picking up a pile of a dozen or two with hands fresh from the patient's mouth, they infect the lot, while trying to separate one. Now Mary can easily at some odd time during the day fold each doily once and by laying it criss-cross of the one below continue till a pile of eighteen or twenty is laid in the box ready for service. One so folded can be readily removed without touching the others.

Then Mary can cut the long absorbent rolls (purchased most economically in boxes of 500) into thirds—the most convenient size. And there is no machine-made pellet that is superior to a good hand-made one. So a few little boxes of these in graded sizes can be made up when Mary's hands are freshly scrubbed.

Some one has said that if there is anything in which a dentist should be extravagant it is clean linen. This can be in Mary's keeping; and if she be a neat needlewoman, she may prevent many a minute wear or tear due to laundry or natural defect from spoiling an otherwise good coat, napkin or towel. By the way, I believe in fair-sized napkins (the style called a tray cloth is convenient in size and form) and in white duck coats. For the latter I may say there is nothing like them in summer and they are neat for winter.

Mary can also be given complete charge of the instruments (except, perhaps, for their sharpening.) After each patient she removes from the bracket table or cabinet shelf all those used and gives them a thorough mechanical cleaning, with a small brush and soap and water, after which they are antiseptically treated, dried and put in place.

If Mary is mechanical by nature it will be a great help. She will then experience little difficulty in telling which way to unscrew the head of the right-angle handpiece at the close of the day, in order to oil it, or in removing and adjusting the saliva-ejector tubes.

### WHY NOT LACTIC?

Some of you have experienced difficulty in keeping the glass tubes clear. I did. So I tried sulphuric, hydrochloric and nitric acids without success. One day my "Mary" said, "why not try lactic?" It was like an inspiration. And I want to show you how it works. Just a drop put in the tube and then swabbed out with a bit of cotton fastened on bind ing wire will do the work.

Another good quality in Mary is a logical mind. Lactic was used in removal of deep seated-salivary calculus; why not use it in dissolving the salivary deposits from the ejector tube?

A few days ago the assistant made a suggestion for improving the rubber polishing cup, and I hope to be able at a meeting in the near future to present as an item of interest a cup that you will all be able to make and use to advantage.

The hypodermic syringe needs attention from time to time. I find it advisable to keep more than one in good working order, and so every week these receive a moistening to keep the plungers snug in the barrel.

### "A BLESSING."

A cool-headed assistant is a blessing in disguise in gas administration. She can prepare the lady patient in the dressing-room and assist in the administration. Then after extraction of any sort the patient can usually be leftimmediately in Mary's care, while the dentist continues other work. After a chloroform case the attendance of the lady assistant is a great comfort. In cases of fainting Mary should know what to do and be prepared to do it. For it is sometimes necessary for both operator and assistant to be at work reviving. Some few months ago I extracted an impacted third molar for a young woman whose mother, a very nervous individual, remained out of the room till the deed was done. When all was completed she was called in. But the sight of her daughter, who was slightly pale, upset her feelings, and reacted upon the daughter. So the assistant was busy with the ammonia in one room, while I found it necessary to give some aromatic spirits in water to the daughter in the chair.

### SAVES TIME.

In gold work the advantage of a trained assistant is most obvious. Mary can save one-third to one-half the time by annealing and passing the gold, while in many places her malleting cannot be equaled by any machine contrivance made.

A considerable saving of time can be made, too, if she will mix cement or amalgam or both while the cavity is being finally sterilized and dried, while in setting of crowns or bridges she is sometimes absolutely indispensable.

Mary can make better gutta-percha points for you than most of the supply houses. She can melt over your old wax on water in dripping pans and cut it into sheets, keep your flasks and vulcanizer in order, start the;vulcanizer, open cases, remove the wax from the water used in separating flasks by using cotton to skim the surface when cooled. Take some of your modeling impressions for orthondontia, pour them, true up the small carborundum stones, polish plates, fashion matrices from broken ribbon saws, watch the electric furnace and examine the supply man's book each day to see that items are correct.

TAKES THE MONEY.

She can save the dentist's hands from soiling by taking all moneys and making the change. She can write the receipts. All credits she will immediately note on the day slip. On this slip she will also note, during or at the close of each operation the patient's name, the service performed, time occupied and generally the charge (if she knows at the time).

At the close of the day the dentist can then use this slip, correcting when necessary, for writing up his cards. I take it for granted that every one now uses a card system of some kind.

It is always advisable to keep in the safe deposit or elsewhere an up-to-date list of accounts that in event of loss of the office ledger may be utilized. Every week this can be corrected by Mary. She will thus become somewhat familiar with the financial side of the office and in a position intelligently to send out on the first of each month such statements as are necessary. No accounts should be sent, of course, with out scanning by the dentist.

SHORT ACCOUNTS.

I am a firm believer in promptly rendered bills. The old saying is, "Short accounts make long friends and long standing ones make short friends." Time effaces the sense of indebtedness. I should advise Mary to make out bills "for professional services," not in detail except by request, which (if we have the patient's confidence) is a thing seldom asked.

Mary can also write tracers if they are necessary, and at dictation such notes as may be further necessary, to follow up accounts. Then she can turn over to the collector such bills as he is to fall heir to. And of all these, statements, notes and delivery to the collector, with any partial or complete payments, she keeps a complete but very simple card record.

Of all important letters she will preserve a copy. Letters from patients, such as may require attention later, she also places on file.

With all the opportunities for service which I have enumerated—and they are by no means the only ones—you will see that Mary's life is a busy one, that she is a woman to be trusted, one whose loyalty is to be desired and to be had if the dentist will prove worthy of it. And I believe she is, like us, able to do better work and more of it with a Saturday half holiday than without it.—DR. R. E. GILSON, *Pacific Gazette.*

